# Development of a Multiplatform Tool for the Prevention of Prevalent Mental Health Pathologies in Adults: Protocol for a Randomized Control Trial

**DOI:** 10.2196/52324

**Published:** 2024-03-11

**Authors:** Nadia Ramos, Felipe Besoain, Natalia Cancino, Ismael Gallardo, Paula Albornoz, Andres Fresno, Rosario Spencer, Soledad Schott, Daniel Núñez, Carolina Salgado, Susana Campos

**Affiliations:** 1 Faculty of Psychology University of Talca Talca Chile; 2 Center of Applied Psychology Faculty of Psychology University of Talca Talca Chile; 3 Faculty of Engineering University of Talca Talca Chile; 4 Doctorate in Psychology Faculty of Psychology University of Talca Talca Chile; 5 Instituto Nacional de Capacitación Talca Chile; 6 Medical School Universidad Catolica del Maule Talca Chile

**Keywords:** adults, anxiety, depression, eHealth, mental health, mobile app, RCT

## Abstract

**Background:**

The prevalence of depression and anxiety has increased in recent years, with many individuals having trouble accessing mental health support. Smartphones have become an integral part of modern life, with apps offering new ways to deliver evidence-based self-help strategies to cope with common mental health symptoms. However, most of them do not have empirical evidence of their overall effectiveness or the effectiveness of their components, which could pose a risk for users.

**Objective:**

The aim of this study is to evaluate the effectiveness of the modules of evaluation, psychoeducation, and emotional regulation strategies in a multiplatform self-help mental health mobile app in the Maule region of Chile.

**Methods:**

A sample of 196 adults will be selected, who will be randomly assigned to different components of the app for a fixed period to assess its ability to reduce symptomatology.

**Results:**

The trial is not yet recruiting and is expected to end in October 2024. The first results are expected in April 2024.

**Conclusions:**

This is the first study in Chile to develop and test the effectiveness of a mobile app to manage anxiety and depression symptoms in adults. The intervention proposed is based on evidence suggesting that the internet or remote intervention tools and self-management of prevalent symptomatology could be the future of mental health care systems in the digital era. If the effects of the intervention are positive, wide implementation in Chile and other Spanish-speaking countries could be possible in the future.

**International Registered Report Identifier (IRRID):**

PRR1-10.2196/52324

## Introduction

### Background and Rationale

Mental health disorders are common in the general population. In Chile, depression and anxiety are the most frequently observed psychopathologies, with a prevalence of 5% for depression and 6.5% for any form of anxiety [1]. However, the population affected by depression or anxiety symptoms may be greater, with 49.2% of Chileans reporting some level of depression symptoms [2]. These prevalences have become a significant issue in public health settings, where waitlists in public mental health services in Chile are mostly stagnant [3], and in the past 2 years have become extensive due to the COVID-19 pandemic. Thus, it becomes necessary to find alternatives that allow providers to respond to this need for mental health access [4].

In this context, the use of remote self-guided psychological interventions, such as those found in mental health mobile apps, has gained popularity [5]. It has been reported that this remote modality improves access, reduces waiting times for appointments, saves time, and reduces the effect of attitudinal barriers in patients seeking psychological support [6]. Studies indicate a high effectiveness rate of web-based care, with smartphone-based psychological interventions reducing user anxiety [7] and depression [8,9]. Accordingly, there has been an increased number of mental health mobile apps available to users. However, many of them do not have sufficient theoretical support for their effectiveness. In 2015, a total of 447 apps related to cognitive behavioral therapy (CBT) were found available in any App Store [10]. Conversely, 9 studies regarding the use of smartphones in cognitive-behavioral interventions were found, of which only 2 had readily available apps in the market [10]. Moreover, 10% of those who say they use cognitive behavioral interventions as a theoretical basis were not evidence-based [11]. It has been suggested that, without empirical support, these apps can be potentially harmful to users [12].

Among the small portion of mobile apps that have empirical support for their tools, 3 specific components or modules have the highest rates of applicability and effectiveness for reducing depression and anxiety: assessment, education, and self-regulation digital tools [13]. The assessment component allows for the recognition of the current symptomatic state of the user, while the constant evaluation helps the monitoring and the development of awareness of the process of change in the symptomatology [14]. The psychoeducation process allows users to be informed about what they are experiencing. Although psychoeducation alone is an effective strategy for the improvement of some anxiety and depression symptoms [15,16], its effectiveness increases when it is accompanied by other strategies [17]. The final component, the use of self-regulation tools, demonstrates a series of brief, easy-to-use self-guided activities that attempt to regulate, express, or otherwise manage psychological distress, low mood, and symptoms of anxiety.

The presentation of these modules normally follows the theoretical framework of CBT. This treatment model can be easily adapted to non–face-to-face environments and has been repeatedly tested in web-based modalities [18,19]. Moreover, other approaches theoretically derived from CBT, such as behavioral activation therapy or mindfulness-based behavioral therapy, also exhibit evidence of their effectiveness in non–face-to-face settings [20]. The behavioral activation treatment for depression attempts to regulate depression symptoms through the programming of pleasurable activities [21], while mindfulness-based CBT focuses primarily on breathing techniques, relaxation, self-compassion, and mindfulness abilities [22], aimed at regulating anxiety states.

According to the literature, there are no mobile apps with scientific support to address depression and anxiety symptoms in the adult population in Chile. The regular and widespread use of such apps could help provide self-care tools to individuals who are reticent about therapy.

### Aims

The main aim of this study is to develop a multiplatform self-help mental health mobile app for adults and to test the effectiveness of its assessment, psychoeducation, and emotional regulation strategy modules post intervention and at 1-month follow-up. We hypothesize that the combination of psychoeducation and any self-regulatory strategies will prove more effective than the evaluation and psychoeducation components alone in decreasing symptomatology.

### Trial Design

This is a protocol for a double-blind, 4-armed randomized controlled trial (ClinicalTrials.gov NCT05997849) evaluating changes in primary and secondary outcomes (symptomatology and well-being variables) post intervention and at follow-up. The 4 arms will be group 1 (control): the participants will have access only to the monitoring and psychoeducation module for 30 days; group 2: participants will have access to the monitoring module, psychoeducation, and mindfulness strategies for 30 days; group 3: participants will have access to the monitoring module, psychoeducation, and behavioral activation strategies for 30 days; and group 4: participants will have access to the monitoring, psychoeducation, and cognitive strategies module for 30 days. Additionally, 1 focus group per condition will be carried out to qualitatively assess the user experience with the app and its overall usability.

## Methods

### Study Setting

Participants will be adults (18 years of age and older) in the Maule Region of Chile and mixed-sex. We expect to recruit 49 participants per arm, configuring 196 participants in total.

### Eligibility Criteria

Adult Chilean citizens, 18 years of age or older, with access to a computer, tablet, or smartphone (Android or iOS) with internet, and no untreated mental health diagnosis, will be included in the study. The mental health diagnosis and current treatment will be assessed solely by the participant’s self-report.

Individuals who self-report substance abuse problems or any current serious mental health disorder and participants reporting scores greater than 1 on question 9 (suicidal ideation) on the Patient Health Questionnaire–9 (PHQ-9) will be excluded from the study.

### Ethical Considerations

This study and all associated documents were approved by the Scientific Ethics Committee of the University of Talca (14/2022). Informed consent will be obtained from all participants through web-based forms before the allocation process begins. The collected data are completely deidentified and stored in secure servers (see Data Management section). Participants will not receive any type of compensation for their participation in the study.

### Recruitment and Informed Consent

For recruitment, invitations will be made through the social networks of the Center for Applied Psychology and the Faculty of Psychology of the University of Talca. In addition, the educational and health services of 4 districts in the region will be contacted to disseminate the multiplatform application Cuidandome to adults. The call for participation will include an explanatory video and an informed consent form, which will be available on and collected through the project website (under development). Participants will be recruited through convenience sampling and will be randomly assigned to 1 of the 4 groups by means of simple randomization (through the PHP: Hypertext Preprocessor rand() function).

### Interventions

#### Description of Interventions

Cuidandome is a multiplatform self-help mental health app that does not constitute a psychological treatment. The mobile app will be available for iOS and Android operating systems, and it is composed of 3 modules: monitoring, psychoeducation, and strategies for people to learn to manage their emotional states and their depression or anxiety symptoms.

The modules of the app are given below.

#### Monitoring (M) Module

The aim of this assessment module is to acquire information on the symptoms and well-being of the participants in the process of using the app and their satisfaction with the strategies. This transversal component is composed of a weekly assessment and a brief daily mood assessment so that the person can monitor their own mood.

#### Psychoeducation (P) Module

The aim of this module is to provide information to the user about different aspects of mental health care, understanding depression and anxiety symptoms, and the fundamentals of the app and how to get the most out of it. Several studies have shown the relevance of psychoeducation in mental health care.

#### Regulation Strategies (RS) Module

**Figure 1:**
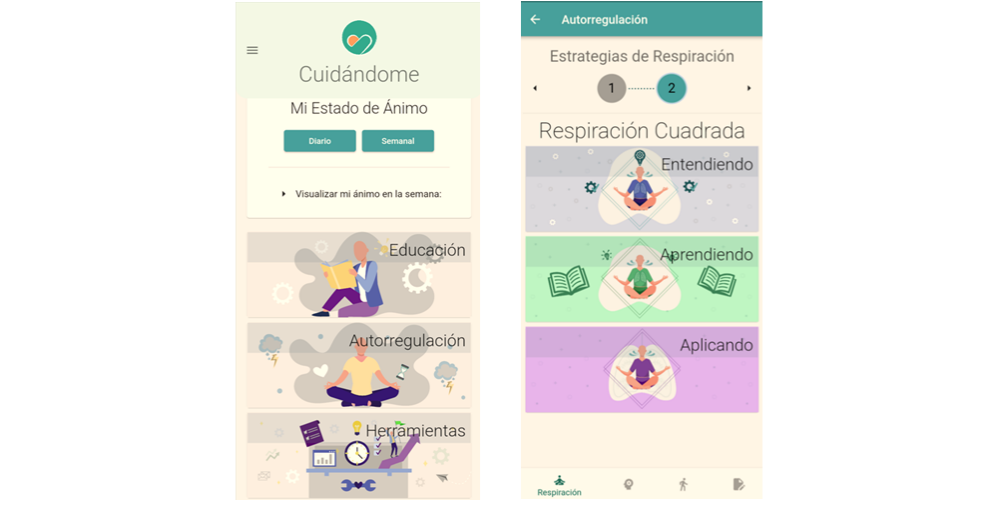
Main dashboard (left) and regulation strategies: square breathing dashboard (right).

This module is based on cognitive behavioral change techniques and strategies originating from the basic principles of CBT for the treatment of depression and generalized anxiety, behavioral activation therapy for depression, and mindfulness strategies for managing anxiety and depression. The module is organized into 3 types of strategies: cognitive (strategies to help modify cognitions that cause discomfort), behavioral activation (strategies focused on modifying behavior that causes discomfort), and mindfulness (strategies to help stay in the present moment and decrease activation of the sympathetic nervous system). Each of the strategies will be developed sequentially through 3 tasks: understanding, learning, and practicing; providing activities to understand why the strategy works and how it should be done; and finally, putting the strategy into practice. Figure 1 shows the overview of the dashboards.

### Criteria for Discontinuing or Modifying Allocated Interventions

Participants in any group can leave the study at any time if they wish without any consequences; this means that their information and collected data will not be analyzed.

### Strategies to Improve Adherence to Interventions

No group will be monitored regarding adherence, as the app is essentially self-guided. However, the app sends daily messages to participants to remind them to access the app and complete at least 1 activity.

### Outcomes

#### Primary Outcomes

Anxiety symptom is 1 of the 2 primary outcomes, and it will be measured by the Generalized Anxiety Disorder–7 Scale (GAD-7). It consists of 7 items that the person assesses on a scale of 0-3 according to the frequency with which each symptom has disturbed them during the past 2 weeks [23]. It shows high reliability in Chilean samples (Cronbach α=0.86 [24]).

Depression symptoms will be measured by the PHQ-9 depression scale, which consists of 9 items that evaluate the presence of depression symptoms present in the past 2 weeks, with a response scale of 0=never, 1=some days, 2=more than half the days, and 3=almost every day. With a Cronbach α of 0.83, its reliability is high in Chilean samples [25]. Additionally, depression and anxiety symptoms in the past week will be assessed through the Patient Health Questionnaire–4 (PHQ-4) [26].

Primary and secondary outcomes will be assessed at 3 time points: preintervention (baseline), immediately post intervention (30 days post intervention), and 1-month follow-up in all groups.

#### Secondary Outcomes

##### Well-Being

Perceived psychological and overall well-being is measured by the Pemberton Happiness Index scale (PHI), a 21-item instrument evaluated in 2 subscales: psychological well-being (experienced 5 positive and 5 negative experiences) and subjective well-being (remembered). The scale is Likert-type, with scores per item from 0 to 10 [27].

##### Resilience

Perceived resilience is measured by the Brief Resilience Scale (BRS), a 6-item instrument on a Likert scale from 1 to 5. It has 3 inverted items, and the rating results from the average of the scores obtained [28].

##### Ruminative Thoughts

Rumination and ruminative thoughts are assessed by the Ruminative Response Scale (RRS, short version), consisting of 10 items that measure ruminant thoughts in 2 dimensions: reflection and restlessness. It has a high level of internal consistency (Cronbach α=0.85). Each item is scored on a 4-point Likert scale from 1 (almost never) to 4 (almost always) [29].

##### Emotional Regulation

Emotional regulation is assessed by the Emotional Regulation Questionnaire (ERQ), a 10-item questionnaire designed to measure respondents’ tendency to regulate their emotions in 2 ways: (1) cognitive reappraisal (1, 3, 5, 7, 8, and 10) and (2) expressive suppression (2, 4, 6, and 9) [30].

##### Mindfulness Skills

Mindfulness skills are assessed with the Mindfulness Attention Awareness Scale (MAAS), an instrument composed of 14 items on a Likert scale from 1 to 6. The scores obtained are added, and the higher the score, the greater the ability to pay attention fully and consciously. The average scores of the nonclinical participants are 65 points out of a total of 84 [31].

### Participant Timeline

See Table 1 for the participant timeline.

**Table 1:** Participant timeline.

Time point			2023												2024																
			Enrollment							Allocation					Post allocation											Close-up					
			September		October		November		December			January		February			March		April		May		June		July			August	September		October
**Enrollment**																															
	Contact with health centers and prospects	✓		✓																											
	Eligibility screen			✓		✓		✓																							
	Allocation							✓			✓																				
	Informed consent										✓		✓																		
**Interventions**																															
	Intervention all arms															✓															
**Assessment**																															
	Baseline variables												✓																		
	Outcome variables															✓		✓		✓											
**Dissemination**																															
	Analyses																					✓		✓			✓				
	Results presentation and publication																												✓	✓	

### Sample Size

To achieve the aims of the study, we have considered 4 arms. To obtain a statistical power of 0.80, a minimum sample size of 36 participants is required for each of the 4 arms. Controlling for a dropout rate of 26%, as shown by the literature on behavioral experiments linked to mobile apps for symptoms related to mood and anxiety [32,33], a final sample size of 49 participants per group is required. The sample size approximation was made based on the literature on statistical power and sample sizes in clinical and social science research [34], while the estimate to reduce the effects of the percentage of abandonment in the experimental conditions was calculated based on Wang and Ji [33]. Thus, an estimate of 196 participants sufficiently obeys such parameters. In addition to the quantitative data collection, a qualitative collection of information will be carried out through focus groups. Regarding the number of focus groups, a total of 4 have been established, 1 for each study group. The size of the focus groups, following the recommendations in the literature [35], is estimated at a total of 32 participants (reaching an estimate of the saturation point), that is, 8 participants per focus group.

### Recruitment

The strategies for achieving adequate participant enrollment to reach the target sample size will include contacting and presenting the study to municipality authorities, who are expected to help establish contact with public health authorities. Interested individuals can sign up to participate through the QR code available in pamphlets delivered to primary health clinics in the Maule area. Health staff in the selected public clinics will also encourage patients to participate in the study.

### Assignment of Interventions: Allocation

#### Sequence Generation

Participants will be randomly assigned to any group with a 1:1 allocation through a website. The web system will generate randomization based on the rand() function of the PHP language. Through this function, a random number between 0 and 3 is generated and subsequently assigned to the participants upon recruitment.

#### Concealment Mechanism

After the randomization and allocation, participants will not receive information regarding the group they belong to. Instead, they will be instructed to access the app and complete the emotional regulation activities, regardless of the type. The participants will only have access to a version of the app that includes the strategies that correspond with their assigned group. Additionally, this information will not be disclosed to the assessment research team (outcome evaluators) to keep the participant allocation blind.

#### Allocation Implementation

Participants will be randomly assigned to any group with a 1:1 allocation through a website. The web system will generate randomization based on the rand() function of the PHP language. Through this function, a random number between 0 and 3 is generated and subsequently assigned to the participants upon recruitment. Depending on the random number assigned, a QR code will be sent to the participants, which will allow them to download a version of the app that contains the strategies pertinent to their study group.

### Assignment of Interventions: Blinding

#### Who Will Be Blinded?

This is a double-blinded trial, blinded to the participants and the research team. A data analyst will work with the final data set, where the group condition will be masked.

#### Procedure of Unblinding If Needed

Unblinding will not occur in this study.

### Data Collection and Management

#### Plans for Assessment and Collection of Outcomes

Self-report questionnaires assessing primary and secondary outcomes will be administered at baseline (immediately before intervention), post intervention, and follow-up. Primary outcomes will be assessed through the website and the app, and secondary outcomes will be measured only on the website.

#### Plans to Promote Participant Retention and Complete Follow-Up

Participants will receive extensive information about the study setup and requirements during the recruitment. This information will include and stress the importance of completion of the follow-up.

#### Data Management

After the participants have completed the web-based questionnaires both on the website and on the app, we will enter the confidential data into a secure platform without identifying information, as each participant will be assigned an encrypted ID number. Only the lead investigator, the research assistants in charge of partial data entry, and the statistician will have access to the complete database. All people with access to the data set will need to sign a confidential agreement to assure the commitment to not reveal identifying information.

### Statistical Methods

#### Statistical Methods for Primary and Secondary Outcomes

We will use descriptive statistics to assess balance across groups at baseline. The primary between-group analysis will be carried out on an intention-to-treat basis for GAD-7 and PHQ-9 scores. We will use a linear mixed model for repeated measures (MMRM) analyses to compare all the intervention and control groups regarding the change in all outcome measures from baseline to post intervention and at follow-up. All the models will be adjusted for sex, age, educational level, and concurrent psychological or psychiatric treatment. Analysis will be carried out with SPSS 26 (IBM Corp) and Stata 15.01 (StataCorp).

#### Statistical Methods for Additional Analyses

Secondary analyses will mainly explore the complex associations between all outcomes. To this end, correlation analyses will be carried out between symptomatic and other psychological variables. Multiple regression analyses and logistic regression analyses among all variables, particularly between mindfulness skills and anxiety symptoms, rumination and depression symptoms, and gender and symptomatology variables will be carried out. Lastly, group comparisons by gender and hours of app use will also be performed. The analysis will be carried out with SPSS 26.

#### Interim Analyses

There will not be interim analyses because the data will be analyzed at the end of the trial.

#### Methods in Analysis to Handle Protocol Nonadherence and Any Statistical Methods to Handle Missing Data

Primary outcomes will be assessed using an intention-to-treat analysis. Missing data will be reduced to a minimum by (1) rendering it impossible to advance on the app if a questionnaire is not completed in full, and (2) using the appropriate measures to encourage participants to fill out the follow-up questionnaires. Multiple imputations will be used to handle any missing data in the analyses.

#### Plans to Give Access to the Full Protocol, Participant-Level Data, and Statistical Code

The data set produced during the study will be available upon reasonable request from the lead researcher NR.

### Oversight and Monitoring

#### Composition of the Coordinating Center and Trial Steering Committee

Daily support for the trial will be provided by the lead investigator, who supervises the trial. Additionally, the study coordinator helps with trial registration and will coordinate and oversee the study visits and reports while organizing data collection and assuring data quality. The data analyst will handle the database and carry out all primary and secondary analyses once all data are collected. Lastly, the app developer will design and implement all content of the app, ensuring the proper functioning of fundamental features such as the randomization of participants, completeness of data collected, automatic creation of the database, etc. The main study team will meet weekly during the duration of the study and monthly with a secondary group of expert collaborators. There is no trial steering committee, stakeholder, or public involvement group. The Ethical Scientific Committee of the Universidad de Talca will check the completeness of the investigation.

#### Composition of the Data Monitoring Committee, Its Role, and Reporting Structure

A monitor from the Ethical Scientific Committee of the Universidad de Talca will check once a year the presence and completeness of the investigation. This committee is independent of the sponsor and has no competing interests. For further details, please contact cec@utalca.cl.

#### Adverse Event Reporting and Harms

The intervention does not cause any harm to the participants. However, if participants experience emotional distress upon using the app, they can contact the lead investigator at any time, who can provide them with options for referral to other public mental health services available. This procedure is explained in detail in the informed consent and initial training videos.

#### Frequency and Plans for Auditing Trial Conduct

A monitor from the Ethical Scientific Committee will check annually the presence and completeness of the investigation files, such as informed consent, inclusion and exclusion criteria, and data collection and storage.

#### Plans for Communicating Important Protocol Amendments to Relevant Parties (eg, Trial Participants and Ethical Committees)

All substantial amendments will be notified to the Ethics Committee of the Universidad de Talca. In case amendments concern or affect participants in any way, they will be informed about the changes. If needed, additional consent will be requested and registered. Additionally, web-based trial registries will be updated accordingly.

#### Dissemination Plans

All results of this research will be disclosed completely in international peer-reviewed journals. Executive summaries of the results will be given to government authorities and public entities acting as stakeholders. Lastly, preliminary relevant results will be presented at an international internet-based mental health seminar organized by the research team, to take place in mid-2024. The full protocol will be made publicly available on the original registry’s website when the study is completed. Publications resulting from this protocol will consider the principal investigators and any participating analyst as authors.

## Results

The trial is not yet recruiting and is expected to end in October 2024. The first results are expected in late April 2024.

## Discussion

### Overview

This study aims to develop a multiplatform self-help mental health mobile app for adults and test the effectiveness of its modules post intervention and at 1-month follow-up. This is the first study in Chile to develop and test the effectiveness of a mobile app to manage anxiety and depression symptoms in adults. The intervention proposed is based on evidence suggesting that the internet or remote intervention tools and self-management of prevalent symptomatology could be the future of mental health care systems in the digital era. If the effects of the intervention are positive, wide implementation in Chile and other Spanish-speaking countries could be possible in the future.

However, this project faces some challenges regarding feasibility and adoption of the intervention, where dropout rates could potentially hinder the results of the experimental procedure, even when accounted for [36]. Additionally, probable limitations are the exclusion of part of the clinical population from the study sample (ie, individuals not currently in treatment or at suicide risk) and the difficulty of controlling the effects of therapy on the primary outcomes in individuals simultaneously undergoing psychotherapy.

### Conclusion

Implementing this research protocol, we expect to provide evidence for the effectiveness of a mental health mobile app in reducing depression and anxiety symptoms in Chilean adults. Moreover, we expect to provide evidence on the associations of these symptom variables and other risk or protective factors for mental health in adults in the context of digital platforms and interventions for health. The potential clinical and research implications derived from this study could help the mental health and well-being of the Chilean population significantly.

## Abbreviations

BRS: Brief Resilience Scale
CBT: cognitive behavioral therapy
ERQ: Emotional Regulation Questionnaire
GAD-7: Generalized Anxiety Disorder-7 scale
MAAS: Mindfulness attention awareness scale
MMRM: mixed model for repeated measures
PHI: Pemberton Happiness Index scale
PHQ-4: Patient Health Questionnaire-4
PHQ-9: Patient Health Questionnaire-9
RRS: Ruminative Response Scale

## Data Availability

The data sets generated and analyzed during this study will be available from the corresponding author upon reasonable request.

## References

[1] (2018). The burden of mental disorders in the region of the Americas. Pan American Health Organization.

[2] Hojman D, Krauze M, Llaupi M, Rojas G, Vergés Á (2018). Resultados Primera Ola Estudio Longitudinal Social de Chile (ELSOC). Módulo 6: salud y bienestar. Salud mental en el Chile de hoy. Notas COES de política pública no.15. Centro de Estudios de Conflicto y Cohesión Social—COES.

[3] Gómez L, Núñez A (2021). Vigilancia del Acceso a la salud en chile: Un sistema de indicadores Para Monitoreo multidimensional. [A system aimed at monitoring healthcare access in Chile]. Rev Med Chil.

[4] Dos Santos Costa AC, Menon V, Phadke R, Dapke K, Miranda AV, Ahmad S, Essar MY, Hashim HT (2022). Mental health in the post COVID-19 era: future perspectives. Einstein (Sao Paulo).

[5] Torous J, Myrick KJ, Rauseo-Ricupero N, Firth J (2020). Digital mental health and COVID-19: using technology today to accelerate the curve on access and quality tomorrow. JMIR Ment Health.

[6] Miralles I, Granell C, Díaz-Sanahuja L, Van Woensel W, Bretón-López J, Mira A, Castilla D, Casteleyn S (2020). Smartphone apps for the treatment of mental disorders: systematic review. JMIR Mhealth Uhealth.

[7] Firth J, Torous J, Nicholas J, Carney R, Rosenbaum S, Sarris J (2017). Can smartphone mental health interventions reduce symptoms of anxiety? A meta-analysis of randomized controlled trials. J Affect Disord.

[8] Bert F, Giacometti M, Gualano MR, Siliquini R (2014). Smartphones and health promotion: a review of the evidence. J Med Syst.

[9] Watts S, Mackenzie A, Thomas C, Griskaitis A, Mewton L, Williams A, Andrews G (2013). CBT for depression: a pilot RCT comparing mobile phone vs. computer. BMC Psychiatry.

[10] Torous J, Levin ME, Ahern DK, Oser ML (2017). Cognitive behavioral mobile applications: clinical studies, marketplace overview, and research agenda. Cogn Behav Pract.

[11] Stawarz K, Preist C, Tallon D, Wiles N, Coyle D (2018). User experience of cognitive behavioral therapy apps for depression: an analysis of app functionality and user reviews. J Med Internet Res.

[12] Baumel A, Torous J, Edan S, Kane JM (2020). There is a non-evidence-based app for that: a systematic review and mixed methods analysis of depression- and anxiety-related apps that incorporate unrecognized techniques. J Affect Disord.

[13] Lagan S, D'Mello R, Vaidyam A, Bilden R, Torous J (2021). Assessing mental health apps marketplaces with objective metrics from 29,190 data points from 278 apps. Acta Psychiatr Scand.

[14] Ritterband LM, Thorndike FP, Cox DJ, Kovatchev BP, Gonder-Frederick LA (2009). A behavior change model for internet interventions. Ann Behav Med.

[15] Carlbring P, Hägglund M, Luthström A, Dahlin M, Kadowaki Å, Vernmark K, Andersson G (2013). Internet-based behavioral activation and acceptance-based treatment for depression: a randomized controlled trial. J Affect Disord.

[16] Qu C, Sas C, Roquet CD, Doherty G (2020). Functionality of top-rated mobile apps for depression: systematic search and evaluation. JMIR Ment Health.

[17] Shen N, Levitan MJ, Johnson A, Bender JL, Hamilton-Page M, Jadad AAR, Wiljer D (2015). Finding a depression app: a review and content analysis of the depression app marketplace. JMIR Mhealth Uhealth.

[18] Andersson G, Hesser H, Veilord A, Svedling L, Andersson F, Sleman O, Mauritzson L, Sarkohi A, Claesson E, Zetterqvist V, Lamminen M, Eriksson T, Carlbring P (2013). Randomised controlled non-inferiority trial with 3-year follow-up of internet-delivered versus face-to-face group cognitive behavioural therapy for depression. J Affect Disord.

[19] Andrews G, Basu A, Cuijpers P, Craske MG, McEvoy P, English CL, Newby JM (2018). Computer therapy for the anxiety and depression disorders is effective, acceptable and practical health care: an updated meta-analysis. J Anxiety Disord.

[20] Mancinelli E, Dell'Arciprete G, Pattarozzi D, Gabrielli S, Salcuni S (2023). Digital behavioral activation interventions during the perinatal period: scoping review. JMIR Pediatr Parent.

[21] Bianchi J, Henao Á (2015). Activación conductual y depresión: conceptualización, evidencia y aplicaciones en Iberoamérica. [Behavioral activation and depression: conceptualization, evidence and applications in Latin America]. Ter Psicol.

[22] Schanche E, Vøllestad J, Visted E, Svendsen JL, Osnes B, Binder PE, Franer P, Sørensen L (2020). The effects of mindfulness-based cognitive therapy on risk and protective factors of depressive relapse—a randomized wait-list controlled trial. BMC Psychol.

[23] Spitzer RL, Kroenke K, Williams JBW, Löwe B (2006). A brief measure for assessing generalized anxiety disorder: the GAD-7. Arch Intern Med.

[24] Crockett MA, Martínez V, Ordóñez-Carrasco JL (2022). Propiedades psicométricas de la escala Generalized Anxiety Disorder 7-Item (GAD-7) en una muestra comunitaria de adolescentes en Chile. Rev Med Chil.

[25] Baader TM, Molina JLF, Venezian SB, Rojas CC, Farías RS, Fierro-Freixenet C, Backenstrass M, Mundt C (2012). Validación y utilidad de la encuesta PHQ-9 (Patient Health Questionnaire) en el diagnóstico de depresión en pacientes usuarios de atención primaria en Chile. Rev Chil Neuro Psiquiatr.

[26] Kroenke K, Spitzer RL, Williams JBW, Löwe B (2009). An ultra-brief screening scale for anxiety and depression: the PHQ-4. Psychosomatics.

[27] Paiva BSR, de Camargos MG, Demarzo MMP, Hervás G, Vázquez C, Paiva CE (2016). The Pemberton Happiness Index: validation of the universal Portuguese version in a large Brazilian sample. Medicine (Baltimore).

[28] Smith BW, Dalen J, Wiggins K, Tooley E, Christopher P, Bernard J (2008). The brief resilience scale: assessing the ability to bounce back. Int J Behav Med.

[29] Treynor W, Gonzalez R, Nolen-Hoeksema S (2003). Rumination reconsidered: a psychometric analysis. Cognit Ther Res.

[30] Gross JJ, John OP (2003). Individual differences in two emotion regulation processes: implications for affect, relationships, and well-being. J Pers Soc Psychol.

[31] Brown KW, Ryan RM (2003). The benefits of being present: mindfulness and its role in psychological well-being. J Pers Soc Psychol.

[32] Torous J, Andersson G, Bertagnoli A, Christensen H, Cuijpers P, Firth J, Haim A, Hsin H, Hollis C, Lewis S, Mohr DC, Pratap A, Roux S, Sherrill J, Arean PA (2019). Towards a consensus around standards for smartphone apps and digital mental health. World Psychiatry.

[33] Wang X, Ji X (2020). Sample size estimation in clinical research: from randomized controlled trials to observational studies. Chest.

[34] Faul F, Erdfelder E, Lang AG, Buchner A (2007). G*Power 3: a flexible statistical power analysis program for the social, behavioral, and biomedical sciences. Behav Res Methods.

[35] Carlsen B, Glenton C (2011). What about N? A methodological study of sample-size reporting in focus group studies. BMC Med Res Methodol.

[36] Hernández-Rodríguez JC, García-Muñoz C, Ortiz-Álvarez J, Saigí-Rubió F, Conejo-Mir J, Pereyra-Rodriguez JJ (2022). Dropout rate in digital health interventions for the prevention of skin cancer: systematic review, meta-analysis, and metaregression. J Med Internet Res.

